# Differential Effects of Indoxyl Sulfate and Inorganic Phosphate in a Murine Cerebral Endothelial Cell Line (bEnd.3)

**DOI:** 10.3390/toxins6061742

**Published:** 2014-06-04

**Authors:** Andréa E. M. Stinghen, Jean-Marc Chillon, Ziad A. Massy, Agnès Boullier

**Affiliations:** 1 Inserm U1088, Department of Pharmacy, 1 rue des Louvels, Amiens F-80037 Cédex 1, France; E-Mails: andreastinghen@ufpr.br (A.E.M.S.); jean-marc.chillon@u-picardie.fr (J.-M.C.); massy@u-picardie.fr (Z.A.M.); 2 Université de Picardie Jules Verne, Amiens F-80025, France; 3 University Medical Center of Amiens, Amiens F-80054, France; 4 Division of Nephrology, Ambroise Paré University Medical Center, Paris, Boulogne Billancourt F-92100, France; 5 Université Paris-Ile-de-France Ouest, Paris, Versailles F-78000, France

**Keywords:** chronic kidney disease, uremic toxins, endothelial dysfunction, nitric oxide, reactive oxygen species

## Abstract

Endothelial dysfunction plays a key role in stroke in chronic kidney disease patients. To explore the underlying mechanisms, we evaluated the effects of two uremic toxins on cerebral endothelium function. bEnd.3 cells were exposed to indoxyl sulfate (IS) and inorganic phosphate (Pi). Nitric oxide (NO), reactive oxygen species (ROS) and O_2_•^–^ were measured using specific fluorophores. Peroxynitrite and eNOS uncoupling were evaluated using ebselen, a peroxide scavenger, and tetrahydrobiopterin (BH_4_), respectively. Cell viability decreased after IS or Pi treatment (*p* < 0.01). Both toxins reduced NO production (IS, *p* < 0.05; Pi, *p* < 0.001) and induced ROS production (*p* < 0.001). IS and 2 mM Pi reduced O_2_•^–^ production (*p* < 0.001). Antioxidant pretreatment reduced ROS levels in both IS- and Pi-treated cells, but a more marked reduction of O_2_•^–^ production was observed in Pi-treated cells (*p* < 0.001). Ebselen reduced the ROS production induced by the two toxins (*p* < 0.001); suggesting a role of peroxynitrite in this process. BH_4_ addition significantly reduced O_2_•^–^ and increased NO production in Pi-treated cells (*p* < 0.001), suggesting eNOS uncoupling, but had no effect in IS-treated cells. This study shows, for the first time, that IS and Pi induce cerebral endothelial dysfunction by decreasing NO levels due to enhanced oxidative stress. However, Pi appears to be more deleterious, as it also induces eNOS uncoupling.

## 1. Introduction

The prevalence of chronic kidney disease (CKD) is increasing exponentially and is closely related to cardiovascular disease (CVD) [1], which accounts for approximately 50% of all deaths in patients with CKD [2,3]. Stroke is the third leading cause of cardiovascular death in these patients, and is five to 10 times more prevalent than in the general population [4]. Moreover, CKD patients have a higher risk of cognitive impairment and dementia, and a poorer long-term prognosis compared to the general population [5]. To date, only a few studies with conflicting results have investigated the risk of cognitive decline, [6,7,8], dementia [9,10] or stroke [11] in CKD patients. The high frequency of stroke and cognitive impairment in CKD patients cannot be explained solely by the high prevalence of traditional risk factors, as emerging non-traditional risk factors such as accumulation of uremic toxins and arterial calcification may also contribute to changes in cerebral perfusion [5].

We recently reported a decrease in endothelium-dependent relaxation in a well-defined murine model of CKD [12] in the absence of any changes in arteriolar structure, mechanics or composition [13]. Endothelial dysfunction is a fundamental step in the atherosclerotic process. It is mainly characterized by decreased bioavailability of endothelium-derived dilators, particularly nitric oxide (NO). In the vasculature, NO is generated by endothelial NO synthase (eNOS) and regulates vascular tone [14]. Multiple factors contribute to decreased NO bioavailability such as L-arginine deficiency, changes in L-arginine transporter, accumulation of endogenous endothelial NO synthase (eNOS) inhibitors (asymmetric dimethylarginine (ADMA) [15], cofactor deficiency (tetrahydrobiopterin or BH4), decreased eNOS gene expression, decreased eNOS mRNA half-life, changes in Gi proteins, changes in calcium-independent pathways of eNOS activation (tyrosine or serine phosphorylation) and changes in the interaction between eNOS and caveolin−HSP90 [16,17]. NO can also be quenched by oxidative stress, increasing the levels of toxic NO-derived species such as peroxynitrite (ONOO^–^) subsequent to reaction of NO with superoxide anion (O_2_•^–^) [18].

Increased oxidative stress induced by reactive oxygen species (ROS) is associated with atherosclerosis and cardiovascular morbidity, not only in the general population but also in CKD patients [19]. The pathogenesis of oxidative stress in CKD patients is multifactorial. It includes several endogenous and exogenous sources such as uremia-related factors, intravenous iron supplementation and dialysis-related factors [20]. Globally, endothelial dysfunction in CKD patients is currently attributed to an imbalance between ROS formation, NO levels and impaired antioxidant defence, which are all common features in these patients [20,21].

To our knowledge, no study has evaluated the impact of uremic toxicity on cerebral endothelial dysfunction, particularly in relation to oxidative stress. As chronic renal failure leads to accumulation of uremic toxins, we investigated the impact of two well-known uremic toxins—indoxyl sulfate (IS) and inorganic phosphate (Pi)—on cerebral endothelial function *in vitro* using the immortalized murine microvascular endothelial cell line (bEnd.3), which is widely used to assess cerebral endothelial function. We investigated whether cerebral endothelial cells release ROS after stimulation by these two uremic toxins and explored the mechanisms partly responsible for the lack of NO bioavailability.

## 2. Results and Discussion

### 2.1. Results

#### 2.1.1. Effect of IS and Pi on Cell Viability

Indoxyl sulfate (IS) is taken up by the cells via several members of the organic anion transporters (OAT) family [22]. Ohtsuki *et al.* have investigated the brain-to-blood transport of IS to clarify the transporter(s) involved in IS transport at the blood brain barrier (BBB) [23]. They have shown that the brain-to-blood transport of IS, an anionic uremic toxin, was mediated by OAT3 [23,24]. Therefore, we first determine OAT3 mRNA expression in our cell line (bEnd.3) by RT-PCR. We found that this cell line expresses OAT3 (data not shown). Similarly, inorganic phosphate is taken up by the cells via phosphate transporters [25] such as Pit-1 and Pit-2 which are ubiquitous expressed. Thus, we checked the mRNA expression of these two transporters in our cell line and found that in fact the bEnd.3 cell line expresses both transporters (data not shown).

**Figure 1:**
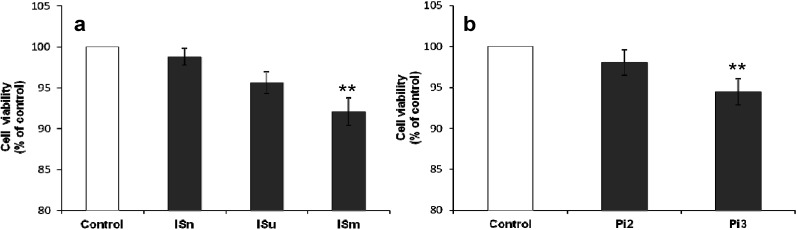
Effect of indoxyl sulfate (IS) (**a**) and inorganic phosphate (Pi) (**b**) on cerebral endothelial cell viability. bEnd.3 cells were incubated with IS or Pi at 37 °C for 24 h, and then treated with 3-(4,5-dimethylthiazol-2-yl)-2,5-diphenyltetrazolium bromide) (MTT) for 3 h. Cell viability was determined by measuring absorbance at 570 nm. Cell viability of untreated control cells was taken as 100%. Data are expressed as mean ± SEM of three independent experiments. ** *p* < 0.01 *vs.* control. ISn, normal IS concentration; ISu, uremic IS concentration; ISm, maximal IS concentration; Pi2, 2 mM Pi; Pi3, 3 mM Pi.

bEnd.3 cell viability was assessed after 24-h incubation with uremic toxins. IS treatment inhibited cell viability in a concentration-dependent manner (Figure 1a), with a slight but significant decrease observed with ISm compared to untreated control cells (−8%, *p* < 0.01). Pi exposure yielded similar results, with a significant decrease of bEnd.3 cell viability observed in response to 3 mM Pi treatment (*p* < 0.01) (Figure 1b).

#### 2.1.2. Effect of IS and Pi on NO Production

**Figure 2:**
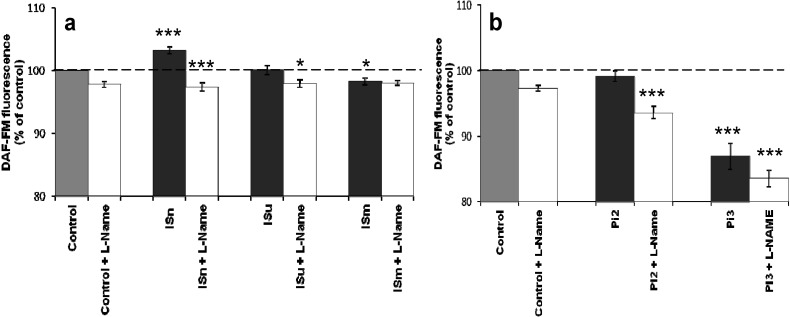
Effect of IS (**a**) and Pi (**b**) on NO production in cerebral endothelial cells. bEnd.3 cells were incubated with 0.1 µM DAF-FM in D-PBS at 37 °C for 1 h and then treated with IS or Pi. NO was determined immediately by measuring fluorescence (λ_Ex_ 492 nm, λ_Em_ 510 nm). NO production in untreated control cells was taken as 100%. Data are expressed as mean ± SEM of four independent experiments. (**a**) * *p* < 0.05—ISm *vs.* Control, ISu + L-Name *vs.* ISu, *** *p* < 0.001—ISn *vs.* Control, ISn + L-Name *vs.* ISn; (**b**) *** *p* < 0.001—Pi2 + L-Name *vs.* Pi2, Pi3 *vs.* Control, Pi3 + L-Name *vs.* Pi3. ISn, normal IS concentration; ISu, uremic IS concentration; ISm, maximal IS concentration; Pi2, 2 mMPi; Pi3, 3 mM Pi; L-Name (*N*-nitro-L-arginine methyl ester), an inhibitor of eNOS activity.

IS treatment showed a biphasic effect: firstly, an increase with ISn (*p* < 0.001) and then a decrease with ISm (*p* < 0.05) (Figure 2a) compared to untreated bEnd.3 cells. Similarly, only the highest concentration of Pi (3 mM) significantly reduced NO production (*p* < 0.001) (Figure 2b). In both sets of experiments (Figure 2a,b), the addition of L-NAME, an inhibitor of eNOS activity, significantly reduced NO levels (*p* < 0.001). Since indoxyl sulfate is mainly bound to albumin in serum, we performed a similar experiment by supplementing the culture medium with albumin at the concentration found in human serum (4%). The presence of albumin did not change the IS effects on NO production (data not shown).

#### 2.1.3. IS and Pi Induce ROS Production

**Figure 3:**
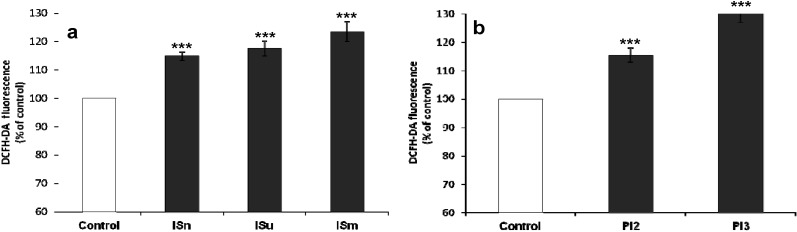
Effect of IS (**a**) and Pi (**b**) on ROS production in cerebral endothelial cells. bEnd.3 cells were incubated with 1 µM DCFH-DA in D-PBS at 37 °C for 30 min and then treated with IS or Pi. ROS was determined immediately by measuring fluorescence (λ_Ex_ 492 nm, λ_Em_ 535 nm). ROS production in untreated control cells was taken as 100%. Data are expressed as mean ± SEM of four independent experiments. *** *p* < 0.001 *vs.* control. ISn, normal IS concentration; ISu, uremic IS concentration; ISm, maximal IS concentration; Pi2, 2 mM Pi; Pi3, 3 mM Pi.

To investigate whether IS and Pi mediated oxidative stress in cerebral endothelial cells, we evaluated their effects on intracellular ROS production using the DCFH-DA probe. Figure 3a shows that IS significantly (*p* < 0.001) and dose-dependently induced ROS production compared to untreated control bEnd.3 cells, with a more marked effect of ISm. Similarly, Pi treatment enhanced oxidative stress as assessed by ROS production, with a peak effect at 3 mM (*p* < 0.001) (Figure 3b).

#### 2.1.4. *N*-Acetylcysteine and α-Tocopherol Decrease Uremic Toxin-Induced ROS Production

**Figure 4:**
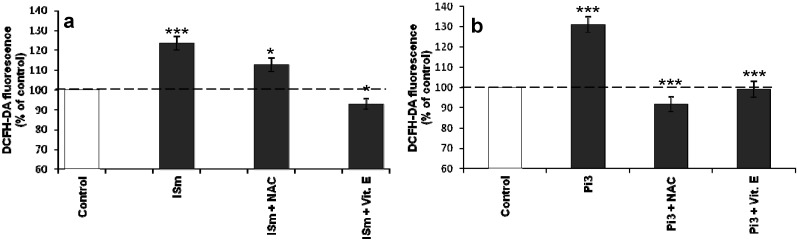
Effect of antioxidants on IS- (**a**) and Pi- (**b**) induced ROS production in cerebral endothelial cells. bEnd.3 cells were incubated with 1 µM DCFH-DA in D-PBS at 37 °C for 30 min and then treated with ISm or 3 mM Pi, with or without the antioxidants α-tocopherol (Vit E, 10 µg/mL) and N-acetyl-L-cysteine (NAC, 10 mM), respectively. ROS levels were determined immediately by measuring fluorescence (λEx 492 nm, λEm 535 nm). ROS levels in untreated control cells were taken as 100%. Data are expressed as mean ± SEM of four independent experiments. (**a**) * *p* < 0.05 *vs.* ISm, *** *p* < 0.001 *vs.* control; (**b**) *** *p* < 0.001—Pi3 *vs.* control, Pi3 + NAC *vs.* Pi3 and Pi3 + Vit E *vs.* Pi3. ISn, normal IS concentration; ISu, uremic IS concentration; ISm, maximal IS concentration; Pi2, 2 mM Pi; Pi3, Pi 3 mM Pi.

To verify whether the addition of antioxidant can reduce the effects of uremic toxins, we pretreated bEnd.3 cells with two antioxidants known to reduce cellular ROS levels, NAC and α-tocopherol. Pre-treatment with either agent reduced ROS levels of cells exposed to either ISm (Figure 4a) or Pi3 (Figure 4b) (*p* < 0.05 and *p* < 0.001, respectively). The effect of α-tocopherol was similar in both groups of treated cells, but NAC was more effective to reduce cellular ROS levels in Pi3-treated cells than in Ism-treated cells.

#### 2.1.5. Effect of IS and Pi on O_2_•^–^ Production

**Figure 5:**
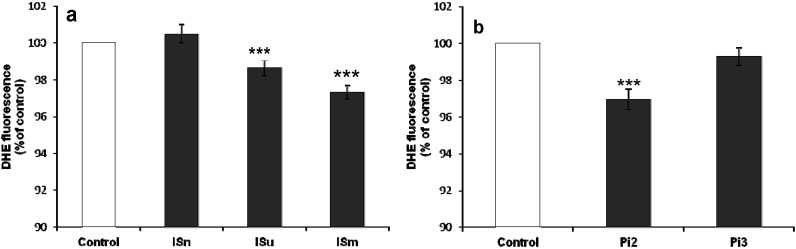
Effect of IS (**a**) and Pi (**b**) on O_2_•^–^ production in bEnd.3 cells. bEnd.3 cells were incubated with 10 µM DHE in D-PBS at 37 °C for 1 h and then treated with IS and Pi. O_2_•^–^ was determined immediately by measuring fluorescence (λ_Ex_ 492 nm, λ_Em_ 615 nm). O_2_•^–^ production in untreated control cells was taken as 100%. Data are expressed as mean ± SEM of four independent experiments. *** *p* < 0.001 *vs.* Control. ISn, normal IS concentration; ISu, uremic IS concentration; ISm, maximal IS concentration; Pi2, 2 mM Pi; Pi3, 3 mM Pi.

Superoxide anion (O_2_•^–^) is an important molecule derived from oxidative stress that has diverse deleterious cellular effects. O_2_•^–^ production was evaluated in bEnd.3 cells treated with IS and Pi. Figure 5a shows that IS induced a slight, but significant concentration-dependent decrease of O_2_•^–^, compared to untreated control cells (*p* < 0.001). A biphasic effect was observed with Pi (Figure 5b), as O_2_•^–^ production was initially decreased with 2 mM Pi (*p* < 0.001), but then increased with 3 mM Pi although it remained lower than the levels observed in control cells.

#### 2.1.6. Effect of Antioxidants on IS- and Pi-Induced O_2_•^–^ Production

**Figure 6:**
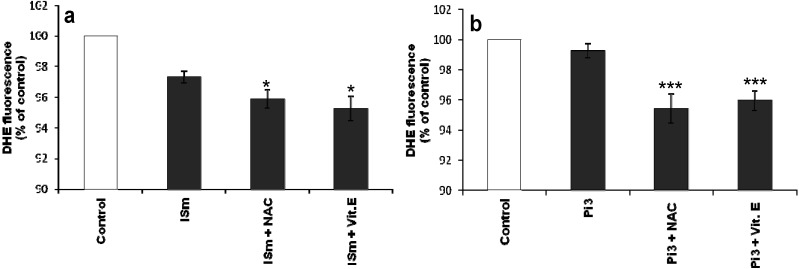
Effect of antioxidants on IS- (**a**) and Pi- (**b**) induced O_2_•^–^ production in bEnd.3 cells. bEnd.3 cells were incubated with 10 µM DHE in D-PBS at 37 °C for 1 h and then treated with IS and Pi with or without the antioxidants α-tocopherol (Vit E, 10 µg/mL) and *N*-acetyl-L-cysteine (NAC, 10 mM). O_2_•^–^ was determined immediately by measuring fluorescence (λ_Ex_ 492 nm, λ_Em_ 615 nm). O_2_•^–^ production in untreated control cells was taken as 100%. Data are expressed as mean ± SEM of four independent experiments. (**a**) * *p* < 0.05 *vs.* IS; (**b**) *** *p* < 0.001 *vs.* Pi3. ISn, normal IS concentration; ISu, uremic IS concentration; ISm, maximal IS concentration; Pi2, 2 mM Pi; Pi3, 3 mM Pi.

Assessment of the effects of antioxidants on uremic toxin-induced O_2_•^–^ production showed that α-tocopherol and NAC were both able to further decrease O_2_•^–^ production in IS- or Pi-treated cells compared to controls (Figure 6a,b). A more marked decrease was observed in Pi-treated cells (*p* < 0.001) than in ISm-treated cells (*p* < 0.05).

#### 2.1.7. Ebselen Reduces Uremic Toxin-Induced ROS Production

**Figure 7:**
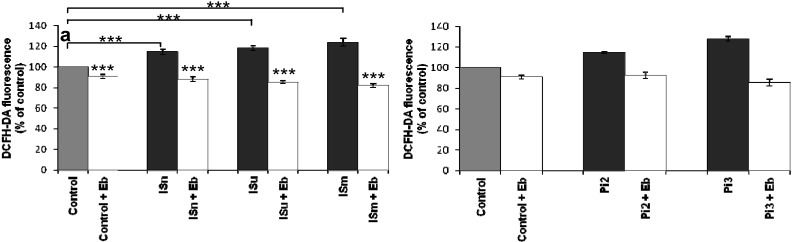
Effect of ebselen on IS (**a**) and Pi (**b**) induced ROS production in bEnd.3 cells. bEnd.3 cells were incubated with 1 µM DCFH-DA alone or with ebselen (10 µM) at 37 °C for 30 min and then treated with IS and Pi. ROS was determined immediately by measuring fluorescence (λ_Ex_ 492 nm, λ_Em_ 535 nm). ROS production in untreated control cells was taken as 100%. Data are expressed as mean ± SEM of four independent experiments. (**a**) *** *P*<0.001—ISn *vs.* Control, ISu *vs.* Control, ISm *vs.* Control, Control + Eb *vs.* Control, ISn + Eb *vs.* ISn, ISu + Eb *vs.* ISu, ISm + Eb *vs.* ISm, Isn *vs.* Control, Isu *vs .* Control, Ism *vs.* Control; (**b**) *** *p* < 0.001—Control + Eb *vs.* Control, Pi2 + Eb *vs.* Pi2, Pi3 + Eb *vs.* Pi3. ISn, normal IS concentration; ISu, uremic IS concentration; ISm, maximal IS concentration; Pi2, 2 mM Pi; Pi3, 3 mM Pi; Eb, Ebselen, a peroxide scavenger.

O_2_•^–^ can react with NO to generate ONOO^–^ which leads to eNOS uncoupling. Therefore, instead of producing NO, uncoupled eNOS generates O_2_•^–^ thereby increasing oxidative stress. To examine the involvement of this process in uremic toxin-induced ROS production, we treated bEnd.3 cells with ebselen, a seleno-organic mimetic of glutathione peroxidase that has been widely used as a scavenger for peroxides such as peroxynitrite. Pretreatment with ebselen significantly reduced enhanced ROS production in response to either uremic toxin towards baseline levels, *i.e.*, the level observed in untreated cells (Figure 7a,b).

#### 2.1.8. Evidence for eNOS Uncoupling

**Figure 8:**
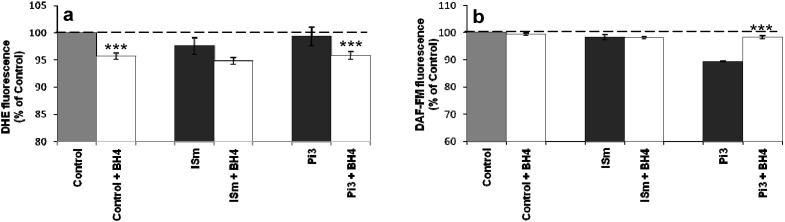
eNOS uncoupling in IS- and Pi- treated bEnd.3 cells. bEnd.3 cells were incubated overnight with 10 µM BH_4_. The cells were subsequently incubated with 10 µM DHE or 0.1 µM DAF-FM in D-PBS at 37 °C for 1 h and then treated with ISm and Pi3. O_2_•^–^ (Figure 8a) and NO (Figure 8b) were determined immediately by measuring fluorescence: λ_Ex_ 492 nm, λ_Em_ 615 nm and λ_Ex_ 492 nm, λ_Em_ 510 nm, respectively. O_2_•^–^ and NO production in untreated control cells were taken as 100%. Data are expressed as mean ± SEM of four independent experiments. (**a**) *** *p* < 0.001—Control + BH_4_ *vs.* Control, Pi3 + BH_4_ *vs.* Pi3. (**b**) *** *p* < 0.001—Pi + BH_4_ *vs.* Pi3. ISm, maximal IS concentration; Pi3, 3 mM Pi; BH4, ((*6R*)-5,6,7,8-tetrahydro-L-biopterin), a redox cofactor of Enos.

As shown above, ROS production was partly mediated by peroxynitrite (Figure 5). Peroxynitrite can mediate oxidation of BH_4_ to BH_2_ leading to uncoupling of eNOS which in turn produces O_2_•^–^ instead of NO and subsequent ONOO^–^ generation. To investigate whether the effects on O_2_•^–^ and NO levels were due to eNOS uncoupling, bEnd.3 cells were pretreated with BH_4_ prior to uremic toxin exposure, and O_2_•^–^ (Figure 8a) and NO (Figure 8b) productions were then determined. The addition of BH_4_ together with IS treatment affected neither NO production nor the amount of O_2_•^–^ produced. On the contrary, a significant increase in NO production (Figure 8b) and a decrease in O_2_•^–^ production (Figure 8a) were observed in Pi-treated cells after addition of BH_4_, suggesting that Pi-treated cells presented a shortage of cofactor BH_4_ that was probably oxidized, leading to eNOS uncoupling.

#### 2.1.9. Nitrotyrosine (NT) Staining of Cells Treated with Uremic Toxins

**Figure 9:**
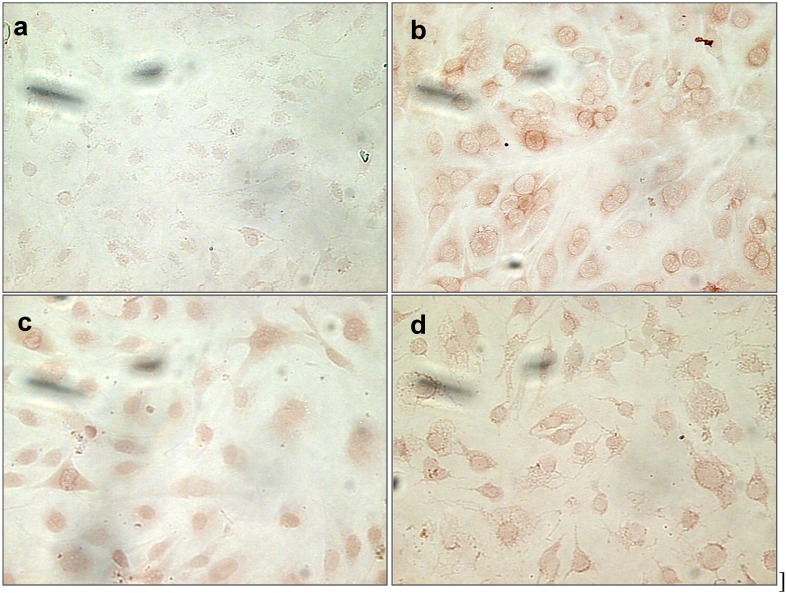
Effect of Pi and IS on immunocytochemical staining for nitrotyrosine in bEnd.3 cells. bEnd.3 cells were treated with IS, Pi or peroxynitrite (500 µM) for 24 h and were stained for nitrotyrosine. Control cells (untreated cells) (**a**); peroxynitrite-treated cells (**b**); ISm- (**c**); and Pi3- (**d**) treated cells. Magnification ×20. ISm, maximal IS concentration; Pi3, 3 mM Pi.

ONOO^–^ reacts with tyrosine and cysteine residues in protein molecules to produce nitrotyrosine and nitrocysteine. Since IS and Pi induce oxidative stress in bEnd.3 cells, we stained treated cells for nitrotyrosine, a marker of oxidative stress. As shown in Figure 9, positive staining was visualized in both ISm- and Pi-treated cells (Figure 9c,d, respectively) compared to untreated cells (Figure 9a). Peroxynitrite-treated cells (Figure 9b) were used as positive control. Staining was specific, as secondary antibody alone did not induce any staining (data not shown). Increased protein nitration was therefore observed after IS and Pi treatment, suggesting an important role of peroxynitrite formation and oxidative stress generation by both IS and Pi.

### 2.2. Discussions

In addition to forming an interface between the intravascular space and the rest of the vessel wall, the endothelium is an important regulator of vascular homeostasis. Endothelial dysfunction is an early marker of atherosclerosis [26] and is predictive of cardiovascular events. One of the most important functions of the endothelium is the synthesis and release of several endothelium-derived relaxing factors such as NO [27]. CKD patients suffer from enhanced atherosclerosis. Numerous studies have demonstrated that endothelial dysfunction occurs in CKD [28] and even begins early in the course of CKD independently of traditional cardiovascular risk factors [29]. A systemic alteration in endothelium-dependent vasodilatation has been known for a long time in CKD patients [30,31]. CKD patients not only experience more atherosclerosis than comparable individuals without kidney disease, but also have a five- to 10-fold higher risk of stroke [4,32]. Using a CKD mouse model, Bugnicourt *et al.* recently demonstrated a decrease in endothelium-dependent vasodilatation of brain arterioles in the absence of any changes in arteriolar structure, mechanics, or composition. They suggested that these alterations in endothelium-dependent relaxation may be partly related to a decrease in NO production [13], but the mechanisms involved in this process are still poorly understood.

As endothelial cells in the presence of uremia are continuously exposed to uremic toxins, the present study investigated whether these toxins could be responsible for the cerebral endothelial dysfunction observed in CKD patients. We observed that two well-known uremic toxins—Pi and IS, whose serum levels are increased in CKD—played a role in this process, based on an *in vitro* model using the murine cerebral endothelial cell line bEnd.3. To our knowledge, this is the first time that the effects of these two toxins on cerebral endothelial cells have been described. The main findings of our study are that: (i) at maximal concentrations, Pi and IS induced oxidative stress as assessed by ROS production; (ii) Pi and IS decreased NO production, partly due to increased oxidative stress; (iii) IS and Pi induced peroxynitrite production; and (iv) only Pi induced eNOS uncoupling, leading to decreased NO production.

As L-arginine was used in all buffers, the observed NO decrease cannot be due to substrate shortage, but rather to decreased production or increased degradation of NO, e.g., resulting from an increase in oxidative stress. Indeed, we observed an increase in ROS production by murine cerebral endothelial cells in response to IS and Pi treatment. It is noteworthy that previous experimental studies have already discussed the role of indoxyl sulfate as an activator of oxidative stress, but this putative role was exclusively based on data obtained in systemic vascular endothelial cells [33,34,35,36]. Although the deleterious effects of hyperphosphatemia can also be partly explained by increased oxidative stress [37,38], only a few experiments have actually demonstrated such an effect in endothelial cells [39,40,41]. Surprisingly, we found that IS and Pi treatment of cerebral endothelial cells led to a decrease in O_2_•^–^ production, in apparent contradiction to the concomitant increase in ROS levels. Since ROS encompass several molecules including free radicals (O_2_•^–^, •OH) and peroxides (H_2_O_2_, ONOO^–^), it is reasonable to suppose that the observed ROS stimulation was due to molecules other than O_2_•^−^. These results could be explained by the fact that, under conditions of oxidative stress such as the uremic state, NO rapidly reacts with O_2_•^–^ to form ONOO^–^. In keeping with this explanation, the addition of ebselen, an ONOO^–^ scavenger, was shown to substantially decrease the DCF signal for both toxins, suggesting an important role of ONOO^–^ in ROS formation by cerebral endothelial cells. Studies by other authors have also shown that IS induces oxidative stress in systemic endothelium *via* NADPH oxidase [33,34,36]. Surprisingly, we did not observe a difference in O_2_•^–^ production elicited by IS in the presence or absence of DPI, an inhibitor of NADPH oxidase (data not shown), suggesting that, in contrast with systemic cells, O_2_•^–^ generation in cerebral endothelial cells is not mainly dependent on NADPH oxidase. Similar results were obtained in response to Pi treatment with or without DPI. These findings are in line with those reported by other authors. Indeed, to determine the potential sources of ROS in bEnd.3 cells, Bevers *et al.* incubated the bEnd.3 cells with apocynin, a NADPH oxidase inhibitor. Since they did not see any difference in the presence or absence of the inhibitor, they concluded that NADPH oxidase, a major source of ROS in systemic endothelial cells, does not contribute to ROS production in bEnd.3 cells [42].

Interestingly, we observed a higher NO production in the presence of normal IS concentration compared to control. From the structural perspective, endogenous indole derivatives could have the potential to function as antioxidants against ROS. In fact, several indole derivatives such as melatonin have been shown to have antioxidants properties [43]. Based on these findings, Miyamoto *et al.* have studied the redox properties of IS, an indole derivative. They have shown that IS at normal concentration exhibit radical scavenging activity, especially against superoxide anion radicals (O_2_•^–^) generated from both a xanthine/xanthine oxidase system and activated neutrophils [44]. Interestingly, they have also shown that while under normal physiological conditions IS seems to play a protective role against oxidative stress, under CKD conditions the pro-oxidant properties of IS exceed its antioxidant properties [45]. Similarly, it has been shown recently that IS could behave as a pro- or anti-oxidant in LDL oxidation models depending of the reactive species involved [46]. O_2_•^–^ can react with NO to generate ONOO^–^ which leads to eNOS uncoupling. Since IS at normal concentration exhibit radical scavenging activity, especially against superoxide anion radicals (O_2_•^–^), it seems reasonable to believe that normal IS concentration is related to higher NO production and therefore could be beneficial.

Another observation of potential interest is that the increase in ROS production by bEnd.3 cells in response to IS exposure was considerably less marked than that previously reported in systemic endothelial cells (HUVEC) [29,30]. This discrepancy could be partly explained by the fact that the cerebral vasculature has several unique features that make it very different from other vascular beds. O_2_•^–^ can be removed enzymatically by superoxide dismutase (SOD) to form the less noxious hydrogen peroxide (H_2_O_2_) [47]. In brain, both manganese- (MnSOD) and zinc-containing SOD (CuZnSOD) are known to effect the conversion of O_2_•^–^ to H_2_O_2_. Cerebral endothelial cells are therefore equipped with a more potent antioxidant defence system comprising of increased SOD activities, compared to systemic endothelial cells [48], making them more resistant to oxidative stress. Antioxidant treatment with NAC or Vitamin E is known to result in decreased oxidative stress. As expected, addition of these antioxidants to the incubation milieu of bEnd.3 cells reduced ROS production in response to the two uremic toxins. However, the antioxidant effect was greater with Vitamin E (α-tocopherol) than with NAC. This difference could be explained by different mechanisms of action. Whereas Vitamin E is a potent radical scavenger but also protects the cell membrane from lipid peroxidation by forming low-reactivity tocopheroxyl radical [49], NAC is a sulfhydryl donor, known to decrease ROS production due to its ability to remove H_2_O_2_, hydroxyl radical and superoxide radicals [50]. Moreover, NAC contributes to the regeneration of glutathione (GSH) [51]. Thiols, including GSH, are thought to play a pivotal role in protecting cells against ROS, and GSH depletion is an established biomarker for oxidative stress. NAC can therefore restore the imbalance between pro-oxidant and antioxidant systems during oxidative stress. The fact that the effect of NAC was greater in Pi-treated cells than in IS-treated cells could result from more ONOO^–^ generation in response to Pi than IS, as peroxynitrite detoxification occurs via reaction with thiol groups such as GSH to form nitrosothiol. Addition of NAC is necessary to regenerate the thiol consumed to detoxify peroxynitrite. S-nitrosothiol is actually a reserve of NO, which can be released in the presence of ascorbate and glutathione peroxidase. Chronic hemodialysis patients have been shown to exhibit not only an increase of plasma S-nitrosothiol levels [52], which could reflect increased peroxynitrite detoxification, but also a decrease in plasma antioxidant levels in favour of more oxidative stress and peroxynitrite. Moreover, the peroxynitrite anion not only decreases O_2_•^–^ detoxification via a rapid reaction between NO and O_2_•^–^, thus driving O_2_•^–^ away from its detoxification system, but can also inactivate human manganese SOD [53], thereby further decreasing O_2_•^–^ detoxification. Once formed, ONOO^–^ reacts with tyrosine and cysteine residues of proteins to produce nitrotyrosine and nitrocysteine. Protein nitration is a common finding in CKD patients and is considered to be a marker of oxidative stress [52,54,55]. In the bEnd.3 cells used in this study, we observed an increase in protein nitration after IS and Pi treatment, suggesting an important role of peroxynitrite formation and oxidative stress generation by both IS and Pi.

Similar results in terms of oxidative stress were observed with IS and Pi. However, our results highlight a major difference between the mechanism of action of Pi and IS. The peroxynitrite anion is a short-lived oxidant species that can promote oxidation of cofactors such as tetrahydrobiopterin (BH_4_) leading to dysfunction of eNOS. Uncoupled eNOS then produces O_2_•^–^ instead of producing NO, in turn increasing oxidative stress. We showed that both toxins induce oxidative stress but in different ways, as addition of BH_4_ restored NO production and decreased O_2_•^–^ only in Pi-treated cells, suggesting that only Pi induces eNOS uncoupling probably by increased oxidative stress and subsequent ONOO^−^ formation.

## 3. Experimental Section

### 3.1. Chemicals and Material

All chemicals were purchased from Sigma (St. Louis, MO, USA) unless otherwise stated.

### 3.2. Endothelial Cell Culture

We used the immortalized murine microvascular endothelial cell line bEnd.3 (CRL-2299, ATCC, Manassas, VA, USA) that is derived from primary mouse brain endothelial cells [56] and widely used for brain studies. This cell line retains key features of differentiated endothelium and is considered to represent an authentic model for examination of cerebral endothelial dysfunction [57]. Cells were cultured in DMEM supplemented with 10% fetal bovine serum (FBS), 2 mmol/L glutamine, 100 IU/mL penicillin, and 100 IU/mL streptomycin, and maintained at 37 °C in a humidified atmosphere containing 5% CO_2_.

### 3.3. Uremic Toxins Preparation

For the concentrations used in our experiments, we referred to the list of uremic toxins provided by the European Uremic Toxin Work Group (EuTox, [58]). Thus, we studied indoxyl sulfate (IS) at normal (ISn—0.6 mg/L–2.3 µM), uremic (ISu—53 mg/L–210.5 µM) and maximum concentrations (ISm—236 mg/L–939.1 µM). Inorganic phosphate (Pi) (NaH_2_PO_4_∙1H_2_O) was used at 1 mM (concentration found in DMEM), 2 mM and 3 mM.

### 3.4. Cell Viability Assay

Cell viability was assessed using the 3-(4,5-dimethylthiazol-2-yl)-2,5-diphenyltetrazolium bromide) (MTT) assay [59]. Briefly, bEnd.3 cells (10^4^ cells/well) were seeded in 96-well plates. After a 24 h incubation, the medium was changed and cells were treated with IS or Pi for 24 h. Culture medium was then replaced with fresh medium (100 µL/well) and 10 µL of MTT solution (5 mg/mL in D-PBS) was added to each well. The plate was left in the incubator for another 4 h. Culture medium was subsequently removed and replaced by dimethylsulfoxide (DMSO) to dissolve the crystals of reduced formazan (700 rpm, 15 min) and absorbance was measured at 570 nm (Envision 2103 Multilabel Reader, Perkin Elmer, CA, USA).

### 3.5. Measurement of NO Production

4-amino-5-methylamino-2’,7’-difluorescein diacetate (DAF-FM) (Life Technologies, Grand Island, NY, USA) was used as a probe to measure NO production. Since NO can react with O_2_•^–^ to form ONOO^–^ and as the main source of O_2_•^–^ in systemic endothelial cells is nicotinamide adenine dinucleotide phosphate-oxidase (NADPH oxidase), diphenyleneiodonium chloride (DPI), an inhibitor of NADPH oxidase, was used to evaluate the involvement of NADPH oxidase. bEnd.3 cells (10^4^ cells/well) were seeded in a white 96-well plate. After 24 h of culture, cells were washed with PBS at 37 °C and labelled with 0.1 µM DAF-FM and 10 µM DPI, an inhibitor of NADPH, for 1h at room temperature. Cells were then washed twice with D-PBS and toxins were added to each well. To examine the specific effect of NO production, 100 µM N-nitro-L-arginine methyl ester (L-NAME), an inhibitor of NO production by eNOS, was added in some experiments. Fluorescence was measured immediately using a spectrofluorimeter (Envision 2103 Multilabel Reader, PerkinElmer, CA, USA) with an excitation wavelength (λ_Ex_ ) at 492 nm and emission wavelength (λ_Em_) at 510 nm. Data were expressed as the percentage increase in fluorescence intensity compared to the control. Some experiments were performed with L-arginine (50 µM) to rule out the possibility that NO decrease was due to reduced substrate levels for eNOS. All solutions were prepared in Krebs-Ringer-Phosphate buffer (KRP) [NaCl 1.5 M; NaH_2_PO_4_ 0.1 M; KCl 0.15 M; MgSO_4_ 0.15 M and CaCl_2_ 0.11 M]. BH_4_ ((*6R*)-5,6,7,8-tetrahydro-L-biopterin) is a redox cofactor of eNOS. As a shortage of BH_4_ can uncouple eNOS which then produces O_2_•^–^ instead of NO, bEnd.3 cells were treated overnight with 10 µM BH_4_ in some experiments.

### 3.6. Measurement of ROS Production

2’,7’-dichlorofluorescein diacetate (DCFH-DA), an ROS-sensitive fluorescent dye, was used to measure ROS production (mostly peroxide). bEnd.3 cells (10^4^ cells/well) were seeded in a white 96-well plate. After 24 h of culture, cells were washed with D-PBS at 37 °C and labeled with 1 µM DCFH-DA for 30 min at 37 °C. Cells were then washed twice with D-PBS and toxins were added to each well. Fluorescence was measured immediately using a spectrofluorimeter (λ_Ex_ 492 nm, λ_Em_ at 535 nm). Data were expressed as the percentage increase in fluorescence intensity compared to untreated cells (control). To evaluate the potential involvement of peroxynitrite (ONOO^−^) in ROS production, cells in some experiments were treated with 10 µM ebselen (2-phenyl-1,2-benzisoselenazol-3 [2H]-one), a lipid-soluble seleno-organic compound that rapidly reacts with peroxynitrite.

### 3.7. Measurement of O_2_•^−^ Production

Under oxidative stress conditions such as uremia, NO can rapidly react with O_2_•^–^ to form ONOO^−^, thereby decreasing the amount of NO available. We therefore measured O_2_•^–^ production using dihydroethidium (DHE) (Life Technologies, Grand Island, NY, USA) as a probe. bEnd.3 cells (10^4^ cells/well) were seeded in a white 96-well plate. After 24 h of culture, cells were washed with D-PBS at 37 °C and labeled with 10 µM DHE for 1h at 37 °C. After two washings with D-PBS, the toxins IS and Pi were added to each well, as previously described. Fluorescence was measured immediately (λ_Ex_ 492 nm, λ_Em_ 615 nm). Data were expressed as the percentage increase in fluorescence intensity compared to the control. In some experiments, bEnd.3 cells were treated with the antioxidants *N*-acetyl-L-cysteine (NAC, 10 mM) or Vitamin E (α-tocopherol, 10 µg/mL) for 1 h prior to treatment with uremic toxins.

### 3.8. Nitrotyrosine Immunostaining

ONOO^–^ can attack proteins, leading to nitrotyrosine formation. We therefore also evaluated nitrotyrosine staining in cells treated by either IS or Pi. bEnd.3 cells (25 × 10^3^ cells/well) were seeded onto coverslips in 24-well plates. When cells had reached about 70% confluence, they were treated with uremic toxins for 1 h, then fixed with 4% paraformaldehyde and permeabilised with 0.1% of Triton X-100 for 10 min. Coverslips were stained with an anti-nitrotyrosine mouse monoclonal antibody (6 µg/mL) (Cayman, Ann Arbor, MI, USA) and then with an anti-mouse HRP-conjugated polyclonal antibody (IgG) (1:500). The reaction was revealed with Vector Red Substrate Kit (Vector Laboratories, CA, USA). Coverslips were mounted on microscope glass slides with Entellan (Merck Millipore, Darmstadt, Germany). Images were captured using a Zeiss fluorescence microscope (Zeiss, Evry, France) system operated by Histolab software (Microvision, Evry, France).

### 3.9. Statistical Analysis

Statistical analysis was performed using JMP Windows version 7.0 (SAS Institute Inc., Cary, NC, USA) and SigmaStat Windows version 3.5 (Systat software Inc., Erkrath, Germany). Data were analyzed by Student’s t test for paired data or Mann-Whitney test and one-way ANOVA for unpaired data. A *p* value < 0.05 was considered significant.

## 4. Conclusions

We hypothesize that both toxins (IS and Pi) contribute to cerebral endothelial dysfunction in CKD patients by increasing oxidative stress and subsequently by reducing NO generation or NO availability *via* two different mechanisms (see Abstract graphic). Moreover, for the first time, we show that, unlike IS, Pi seems to be more deleterious for cerebral endothelial cells, as it leads not only to increased ROS production but also to eNOS uncoupling. However, further investigations such as signalling experiments will be necessary to elucidate the molecular mechanisms involved. Given the high prevalence of brain disease such as stroke in CKD population, a better understanding of the interactions between renal impairment, *i.e.*, uremic toxin accumulation and brain function, could help to minimize progression of brain diseases in CKD patients.
